# The current state of public health education in India: A scoping review

**DOI:** 10.3389/fpubh.2022.970617

**Published:** 2022-11-23

**Authors:** Ashish Joshi, Ashruti Bhatt, Mansi Gupta, Ashoo Grover, Sofia Rani Saggu, Isha Vikas Malik

**Affiliations:** ^1^Graduate School of Public Health and Health Policy, City University of New York, New York, NY, United States; ^2^Foundation of Healthcare Technologies Society, New Delhi, India; ^3^Division of Non Communicable Diseases, Indian Council of Medical Research, New Delhi, India

**Keywords:** public health, public health education, public health management cadre, India, Ph.D., BPH/BPHSc, MPH, Postgraduate Diploma

## Abstract

With the creation of public health management cadre in the state, district, and block levels of India, there is a need for a comprehensive, synergistic education system to ensure efficient public health across the country. This scoping review, therefore, aims to examine the characteristics of public health education programs available in India's varied geographical and regional contexts. It examines 16 program-related descriptors across public health Doctoral, Masters, Bachelors, Post-graduate Diploma, and Diploma education programs offered. Data was retrieved through institutional websites. Results of our analysis showed 84 unique institutions in 20 states and 3 UTs currently offering 116 public health programs across India's 28 states and 8 UTs. Private and public institutes were 65% (*n* = 75) and 35% (*n* = 41) respectfully. The majority of universities mainly provided Masters of Public Health (*n* = 73, 63%) programs followed by Postgraduate Diploma (PGD) and Diploma (*n* = 17, 15%), BPHSc (*n* = 14, 12%), and Ph.D. (*n* = 12, 10%). The majority of Ph.D. programs in public health are offered in Maharashtra, Karnataka, and Haryana, while Masters in Public Health programs are offered highest in Karnataka, Bachelors in Public Health programs in Rajasthan, Post Graduate Diploma in Public Health program in Delhi, and Tamil Nadu had the most number of Diploma in Public Health programs. Thirty-one percent (*n* = 36) of the public health programs are offered across the south, 28% (*n* = 32) across the north, and 22% (*n* = 26) across the west Analyzed descriptors provide comprehensive information on program characteristics, mainly admission, format, and tuition fee. The review offers five suggestions to improve collaborative public health education and prepare a workforce with the skills, knowledge, and expertise to respond to the twentyfirst century's public health threats and challenges in India.

## Introduction

The COVID-19 pandemic continues to be a major public health problem globally. The public health workforce has been undermined by decades of underinvestment. State and local health departments across countries have failed to attract, recruit and retain skilled health professionals required to respond to health threats due to inadequate funding. Lagging skills among workers due to changing technology, lack of systems and data to assess the existing gaps for the required workforce, and various hiring barriers that exist at federal, state, and local levels have resulted in several challenges affecting workforce development. There is a need for pro-activeness in planning for tomorrow's workforce through today's actions. Of the various lessons taught by COVID-19, the need for investment in a robust public health workforce has come up strongly (1).

One of the six building blocks of the World Health Organization (WHO's) framework for health is a strong health workforce. There is a need for health professionals with sound technical knowledge (2).

The current pandemic has its own novelty and scope which has exposed a gap in the competency and the surge capacity of the public health workforce (3). The COVID-19 pandemic has brought the public health workforce in limelight. A significant shift was observed in the years prior to the pandemic across various specializations of the public health workforce. The pandemic has increased the calling for epidemiologists and statisticians to play a leading role in COVID-19 response (4). There is a need for capacity building and identifying gaps in academic programs and the requirements of employers. An adequately trained workforce protects and promotes health, advocates for disparities, and responds rapidly to various health challenges (5).

India with a 1.3 billion population, is second-largest in the world and spread over 3,287,259 km^2^ making it the seventh-largest country in the world. The population is approximately one-sixth of the world's population (6, 7). In India, understaffed health systems, inadequate distribution of qualified workers, workforce shortages, and undersubscribed training programs along with an inadequate number of workforce and workers with a limited appropriate mix of skills have always been a challenge in solving complex health system challenges (8). Many nationwide programs to control or eliminate diseases have been established in the country. Public health in India is growing at a relatively slow rate. Adequate public health workforce training with the required skills to address complex public health challenges is the need of the hour, however, insufficient national standards for public health education, including curriculum and methods have resulted in a limited public health workforce (9). This situation results in ill-equipped public health workers with insufficient competencies, not online in the medical and nursing disciplines, but also among public health engineers, veterinarians specializing in public health, social scientists working in public health, statisticians working with public health-related databases, health workers and ground-level workers such as Accredited Social Health Activists (ASHAs), who are responsible for health promotion, health education, and other key responsibilities (10).

India's health care delivery system faces multiple shortages in the backdrop of poor health indicators across the country. The existing workforces that do not have requisite training are loaded with managerial functions. This needs to be replaced by a professional public health managerial cadre to ensure a safe, effective, and accountable health system. The extent to which public health can be enhanced is mainly determined by the quality of the public health workforce, which is determined by the relevance and quality of its training and educational opportunities (10). The availability of multidisciplinary public health professionals would enhance the equity and efficiency of healthcare delivery and would also relieve the burden on clinical professionals to cope with managerial functions. There are extremely few public health institutes in India, and national requirements for public health education are inadequate This situation results in ill-equipped public health workers with inadequate competencies, not only from the nursing and medical disciplines, but also from public health engineers, veterinarians specializing in public health, social scientists employed in community health, data analysts working with public health related database systems, health workers and ground level workers such as Accredited Social Health Activists (ASHAs), who are responsible for health promotion, health education, and many other responsibilities (10). The High-Level Expert Group, (HLEG) emphasized the need for the establishment of public health institutes across India (11). The National Health Policy 2017 envisages the creation of a Public Health management cadre in all states. The learning from the COVID-19 pandemic highlighted the need for a robust Public Health cadre. The surveillance and expert committee were held in 2020 wherein members participated and endorsed the creation of the Public Health Management cadre.

At the state, district, and block levels, the health ministry has recommended four verticals: specialist cadre, public health cadre, health management cadre, and teaching cadre. This highlights the need for a comprehensive synergistic system to ensure efficient public health in India. The most important challenge is to develop a framework that facilitates such public health management cadre to be accepted into the system (12).

Recent studies have reviewed public programs such as Masters in public health, Bachelors in public health, and certificate and diploma courses across the country. The program format and offerings have changed over the years as institutes try to deliver programs of high quality. Seminal contributions have been made by recent literature in charting public programs across India. Health education developments, notably the establishment of departments of preventive and social medicine in medical schools and health electives in other courses such as social work, enhanced India's ability for studying health inequalities (13). As public health is multidisciplinary, the Medical colleges in India only include public health as part of curriculums but do not have independent departments (14). Several authors have recognized the growing public health sphere and the need to meet the qualitative demand (15–17). More recent examples of studies in the arena of public health education in India can be found in the works of public health faculties in India (17–19), however their methodological approaches vary. The studies discuss competencies and course curriculums and do not capture the geographical distribution across India.

There has been a rapid increase in the number of public health programs offered in India given the increased demand for the public health workforce. Several institutes offer public health programs but due to a lack of council for regulating the programs, there is limited information available about these programs. Analysis of the various public health programs in India will help understand the public health education landscape in India.The study's objectives are to examine the characteristics of public health programmes offered across India. The study will evaluate program-related descriptors such as mode of delivery, program duration, program fee, program content, internship opportunities, scholarship, and competencies that students acquire to prepare this workforce that has the skills, knowledge, and expertise to respond to the public health emerging threats and challenges of the twentyfirst century.

## Methodology

### Search methodology

A search was conducted from August 2021 to November 2021 using the “Google search engine”. The keywords used for the search are as follows; “Public health education” AND, “public health training,” OR “BPH in India,” OR “Bachelors in Public health programs in India,” OR “MPH in India,” “Masters in Public health in India,” OR “Ph.D. in public health in India,” OR “Diploma in Public health in India” OR “Postgraduate diploma in public health in India,” AND/OR “public health universities and institutes.” The search results showed the website links of the institutes offering the courses and various third-party websites and, career counseling websites showing a listing of respective programs offered. The first 20 result pages were adapted in our study. The listing of institutes and programs was done by referring to these websites. A centralized database of public health programs was developed, and duplicates were removed and filtered through a criteria (Appendix 1). The inclusion criteria included only those public health programs to be included in the analysis that was offered by a recognized institute or a University. Third-party websites, links or proxy websites, and websites with missing and incomplete information were excluded. Institutes that no longer offered the program were excluded.

### Variable extraction

Each program website was carefully studied to extract the variables of interest. These 16 variables have been listed and described in Table 1.

**Table 1:** Variable description.

**S.no**	**Data variable**	**Description**
1.	University name	Name of the university offering the public health training program.
2.	Institute name	Name of the institute offering the program.
3	State/Union territory	The State/Union territory in which the Institute/University offering the public health program is located.
4.	City	The city in which the Institute/University offering the public health program is located.
5.	Institute type	Institutes offering public health programs classified as Public or Private institutes.
6.	Official website	The official website of the public health program being offered by the Institute/University.
7.	Programs offered	The information was recorded on the various public health programs offered by the Institute/University.
8.	Program fees or tuition	Total fees for the various public health programs offered by the institute/University.
10.	Eligibility criteria	Prerequisites for the various public health programs recorded.
11.	Admission procedure	The process of admission and whether the candidate needs to appear for an exam, interview, or group discussion.
12.	Mode of delivery of the program	Whether the program is conducted online, on-campus, in hybrid mode, full-time, or part-time.
13.	Internship requirement	Whether there is an internship requirement for completion of the program and its tenure is mentioned.
14	Specializations	Whether there are any specializations offered.
15	Fees	Averages fees per semester/year as provided.
16	Course duration	Period of offering the course in years.

## Results

### Overview of public health programs across Indian states and UTs

**Figure 1:**
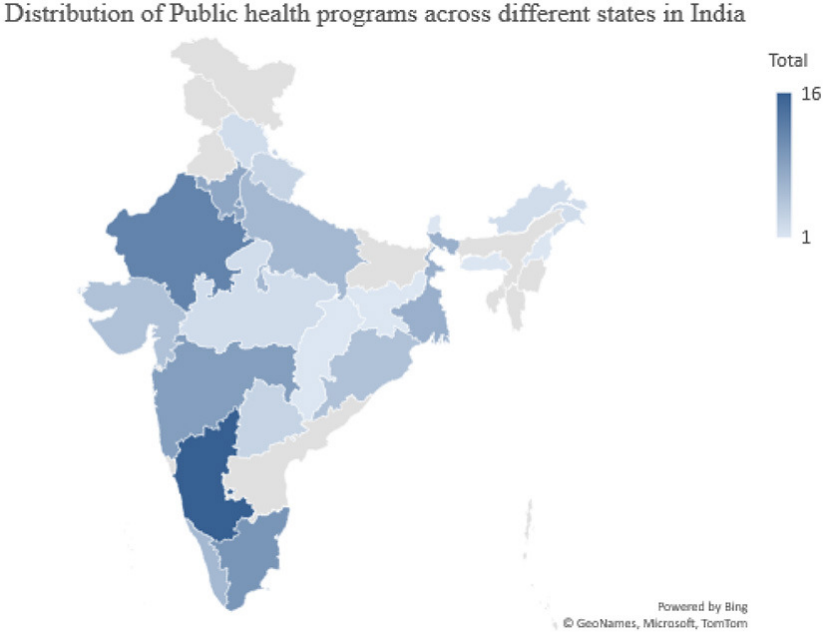
Distribution of public health programs across different states in India.

Out of all the 28 states and 8 UTs of India, 84 unique universities in 20 states and 3 UTs are providing 116 Public health programs (Figure 1; Table 2). The majority of universities mainly provided Masters of Public Health (*n* = 73, 63%) programs followed by Postgraduate Diploma (PGD) and Diploma (*n* = 17, 15%), BPHSc (*n* = 14, 12%), and Ph.D. (*n* = 12, 10%). The eight states including Andhra Pradesh, Assam, Bihar, Goa, Manipur, Mizoram, Punjab, Tripura, and five UTs Andaman and Nicobar Island, Dadra and Nagar Haveli & Daman and Diu, Jammu and Kashmir, Lakshadweep, and Leh-Ladakh do not have any public health programs. States like Karnataka, Rajasthan, and Tamil Nadu offered most of the public health programs. Fifty percent of the doctoral programs are offered each by both the public (*n* = 6) and private Universities (*n* = 6). While in the case of master's in public health programs (*n* = 73), the majority of them (74%; *n* = 54) are offered by the private institutes compared to 26% (*n* = 19) that are offered by public institutes. In the case of bachelor's programs in public health (*n* = 14), 78% (*n* = 11) were offered by private universities compared to 21% (*n* = 3) of them that were offered by public institutes. Eighty-six percent of the (*n* = 12) postgraduate diploma program in public health is offered by public universities and only 14% (*n* = 2) of the private universities offered postgraduate diploma in public health programs. Similarly, the diploma program (*N* = 3) is only offered by one public university and two private universities.

**Table 2:** List of unique institutes/universities offering public health programs across India (*n* = 84).

**Institute/University name**	**Region**	State^*^	City^*^	**Public/private**	**Ph.D**.	**MPH**	**BPHSc**	**PGD**	**Diploma**
Academy of scientific and innovative research	Northern	Delhi	Delhi	Private	No	Yes	No	No	No
AIPH University	Eastern	Odisha	Bhubaneshwar	Private	Yes	Yes	No	No	No
Amity University	Northern	Haryana/Uttar Pradesh^*^	Gurugram/Noida^*^	Private	Yes	Yes	No	No	No
Amrita Vishwa Vidyapeetham	Southern	Kerala	Cochin	Private	No	Yes	No	No	No
Government Medical University	Eastern	West Bengal	Kolkata	Public	No	No	No	Yes	No
Arunodaya university	North-Eastern	Arunachal Pradesh	Itanagar	Private	No	Yes	No	No	No
Chitkara University	Northern	Chandigarh	Chandigarh	Private	No	Yes	No	No	No
Apex University	Western	Rajasthan	Jaipur	Private	No	No	Yes	No	No
Christian Medical College (affiliated to Shree Chitra Tirunal Institute for Medical Sciences and Technology)	Southern	Tamil Nadu	Vellore	Private	No	Yes	No	No	No
Career Point University	Western	Rajasthan	Alaniya/Kota^*^	Private	No	Yes	Yes	No	No
Datta Meghe Institute Of Medical Science	Western	Maharashtra	Wardha	Private	No	Yes	No	No	No
Post Graduate Institute of medical education and research	Northern	Chandigarh	Chandigarh	Public	No	No	No	Yes	No
DY Patil University	Western	Maharashtra	Pune	Private	No	Yes	No	No	No
Eternal University	Northern	Himachal Pradesh	Baru Sahib/Sirmaur	Private	Yes	Yes	No	No	No
G D Goenka University	Northern	Haryana	Gurugram	Private	No	Yes	No	No	No
Galgotias University	Northern	Uttar Pradesh	Greater Noida	Private	No	Yes	No	No	No
Gurugram University	Northern	Haryana	Gurugram	Private	No	Yes	No	No	No
Himalyan Garhwal University	Northern	Uttarakhand	Garhwal	Private	No	Yes	No	No	No
Indian School of Business Management And Administration	Southern	TamilNadu	Chennai	Private	No	Yes	No	No	No
Johns Hopkins University	Western	Rajasthan	Jaipur	Private	No	Yes	No	No	No
JSS University	Southern	Karnataka	Mysore	Private	No	Yes	No	No	No
Kaloji Narayana Rao University of Health Sciences, Warangal	Southern	Telangana	Hyderabad	Private	No	Yes	No	No	No
Post graduate institute of medical sciences	Northern	Punjab/Haryana^*^	Chandigarh	Public	Yes	No	No	No	No
Institute of Public Health	Southern	Karnataka	Bengaluru	Private	Yes	No	No	No	No
Krishna Institute of Medical Sciences	Central	Madhya Pradesh	Chhatarpur	Private	No	Yes	No	No	No
Manipal University	Southern	Karnataka	Mangalore	Private	No	Yes	No	No	No
Maulana Abul Kalam Azad University of Technology, West Bengal	Eastern	West Bengal	Kolkata/Haldia	Private	No	Yes	No	No	No
MIT World Peace University	Western	Maharashtra	Pune	Private	No	Yes	No	No	No
NITTE Education Trust	Southern	Karnataka	Mangalore	Private	No	Yes	No	No	No
NM (University partners)	Not mentioned	Multiple locations	Multiple locations	Private	No	Yes	No	No	No
Noida International University	Northern	Uttar Pradesh	Gautam Buddha Nagar	Private	No	Yes	No	No	No
SRM Institute of Science and Technology	Southern	Tamil Nadu	Chennai	Private	Yes	No	Yes	No	No
Indira Gandhi open university	Northern	Delhi	Delhi	Public	No	No	No	Yes	No
North East Frontier Technical University	North-Eastern	Arunachal Pradesh	Aalo	Private	No	Yes	No	No	No
NSHM College Of Management And Technology, Kolkata	Eastern	West Bengal	Kolkata	Private	No	Yes	No	No	No
Pavara institute of medical science	Western	Maharashtra	Ahmednagar	Private	No	Yes	No	No	No
Poornima University	Western	Rajasthan	Vidhani	Private	No	Yes	No	No	No
Om Sterling Global University	Northern	Haryana	Hisar	Private	No	Yes	Yes	No	No
Parul University	Western	Gujarat	Vadodara	Private	No	Yes	Yes	No	No
Poornima University School of Public Health	Western	Rajasthan	Jaipur	Private	No	Yes	Yes	No	No
Rajiv Gandhi University Of Health Sciences	Southern	Karnataka	Bangalore	Private	No	Yes	Yes	No	No
Ramaiah University	Southern	Karnataka	Banglore	Private	No	Yes	No	No	No
Sai Business And Media School, Sai Group Of Institutions	Northern	Uttarakhand	Dehradun	Private	No	Yes	No	No	No
SGT University	Northern	Haryana	Gurugram	Private	No	Yes	No	No	No
SRM University	North-Eastern	Sikkim/Tamil Nadu^*^	Gangtok/Kattankalathur^*^	Private	No	Yes	No	No	No
Lachoo Memorial College of Science and Technology	Western	Rajasthan	Jodhpur	Private	No	No	Yes	No	No
Sushant University	Northern	Haryana	Gurugram	Private	No	Yes	No	No	No
Symbiosis University	Western	Maharashtra	Pune	Private	No	Yes	No	No	No
Mahatma Jyoti Rao Phoole University	Western	Rajasthan	Jaipur	Private	No	No	Yes	No	No
Ministry of health and family welfare	Northern	Delhi	Delhi	Public	No	No	No	Yes	No
Indian Institute of skill development	Not mentioned	Not mentioned	Not mentioned	Public	No	No	No	Yes	No
Martin Luther Christian University	North-Eastern	Meghalaya	Shillong	Private	No	No	Yes	No	No
Vocational Institution of Ministry of HRD, Government of India (AVI no-710367) and all the courses of IGMPI are approved for lifetime empanelment under Ministry of Horticulture and Food Processing, Government of Uttar Pradesh	Not mentioned	Not mentioned	Not mentioned	Public	No	No	No	Yes	No
The Global Open University	North-Eastern	Nagaland	Dimapur	Private	No	Yes	No	No	No
The Y.B.*N* University	Eastern	Jharkhand	Ranchi	Private	No	Yes	No	No	No
University Of Technology	Western	Rajasthan	Jaipur	Private	No	Yes	No	No	No
Vinayaka Missions University	Southern	Tamil Nadu	Salem	Private	No	Yes	No	No	No
Yenepoya University	Southern	Karnataka	Mangalore	Private	No	Yes	Yes	No	No
All India Institute of Medical Sciences	Western/Northern/ Central	Rajasthan/ Uttarakhand/ Chattisgarh^*^	Jodhpur/Rishikesh/ Raipur^*^	Public	No	Yes	No	No	No
Central University Of Kerala	Southern	Kerala	Kasaragod	Public	No	Yes	No	No	No
Singhania University	Western	Rajasthan	Jhunjhunu	Public	No	No	Yes	Yes	No
Delhi Pharmaceuticals Sciences And Research University	Northern	Delhi	Delhi	Public	No	Yes	No	No	No
Guru Gobind Singh Indraprastha University	Northern	Delhi	Delhi	Public	No	Yes	No	No	No
James Lind Institute	Southern	Karnataka	Bengaluru	Private	No	No	No	Yes	No
Indian Council of Medical Research and NIE	Southern	TamilNadu	Chennai	Public	No	Yes	No	No	No
Jamia Hamdard University	Northern	Delhi	Delhi	Public	No	Yes	No	No	No
Jawahar Lal Nehru University	Northern	Delhi	New Delhi	Public	No	Yes	No	No	No
Jawaharlal Institute of Postgraduate Medical Education and Research	Southern	Pondicherry	Pondicherry	Public	No	Yes	No	No	No
KLE Academy of Higher Education and Research	Southern	Karnataka	Belagavi	Public	Yes	Yes	Yes	No	No
Kalinga Institute of Industrial Technology	Eastern	Odisha	Bhubaneshwar	Public	No	Yes	No	No	No
The Tamil Nadu Dr. M.G.R Medical University	Southern	Tamil Nadu	Not mentioned	Private	No	No	No	No	Yes
Karnataka State Rural Development And Panchayat Raj University	Southern	Karnataka	Bengaluru	Public	No	Yes	No	No	No
Kerala University of Health Sciences	Southern	Kerala	Kottayam/ Thiruvananthapuram^*^	Public	No	Yes	No	No	No
NIMHANS	Southern	Karnataka	Bengaluru	Public	No	Yes	No	No	No
Sree Chitra Tirunal Institute for Medical Sciences and Technology	Southern	Kerala	Trivandrum	Public	Yes	Yes	No	No	Yes
Punjab University	Northern	Chandigarh	Chandigarh	Public	No	Yes	No	No	No
Sam Higginbottom University Of Agriculture, Technology And Sciences	Northern	Uttar Pradesh	Allahabad	Public	No	Yes	No	No	No
Tata Institute of Social Sciences	Western	Maharashtra	Mumbai	Private	Yes	Yes	No	No	No
Sri Ramchandra Institute Of Medical Sciences	Southern	Tamil Nadu	Chennai	Public	No	Yes	No	No	No
University Of Hyderabad	Southern	Telangana	Hyderabad	Public	No	Yes	No	No	No
University Of Lucknow	Northern	Uttar Pradesh	Lucknow	Public	No	Yes	No	No	No
West Bengal University of Health Sciences	Eastern	West Bengal	Kolkata	Public	No	Yes	No	Yes	Yes
Public health foundation of India	Western	Gujarat/Delhi^*^	Gandhinagar/Delhi^*^	Public	Yes	No	No	Yes	No
Savitribai Phule Pune University	Western	Maharashtra	Pune	Public	Yes	Yes	No	No	No

* “/” Signifies that the public health programs are available at more than one location for that university.

#### Geographical distribution of public health programs

**Figure 2:**
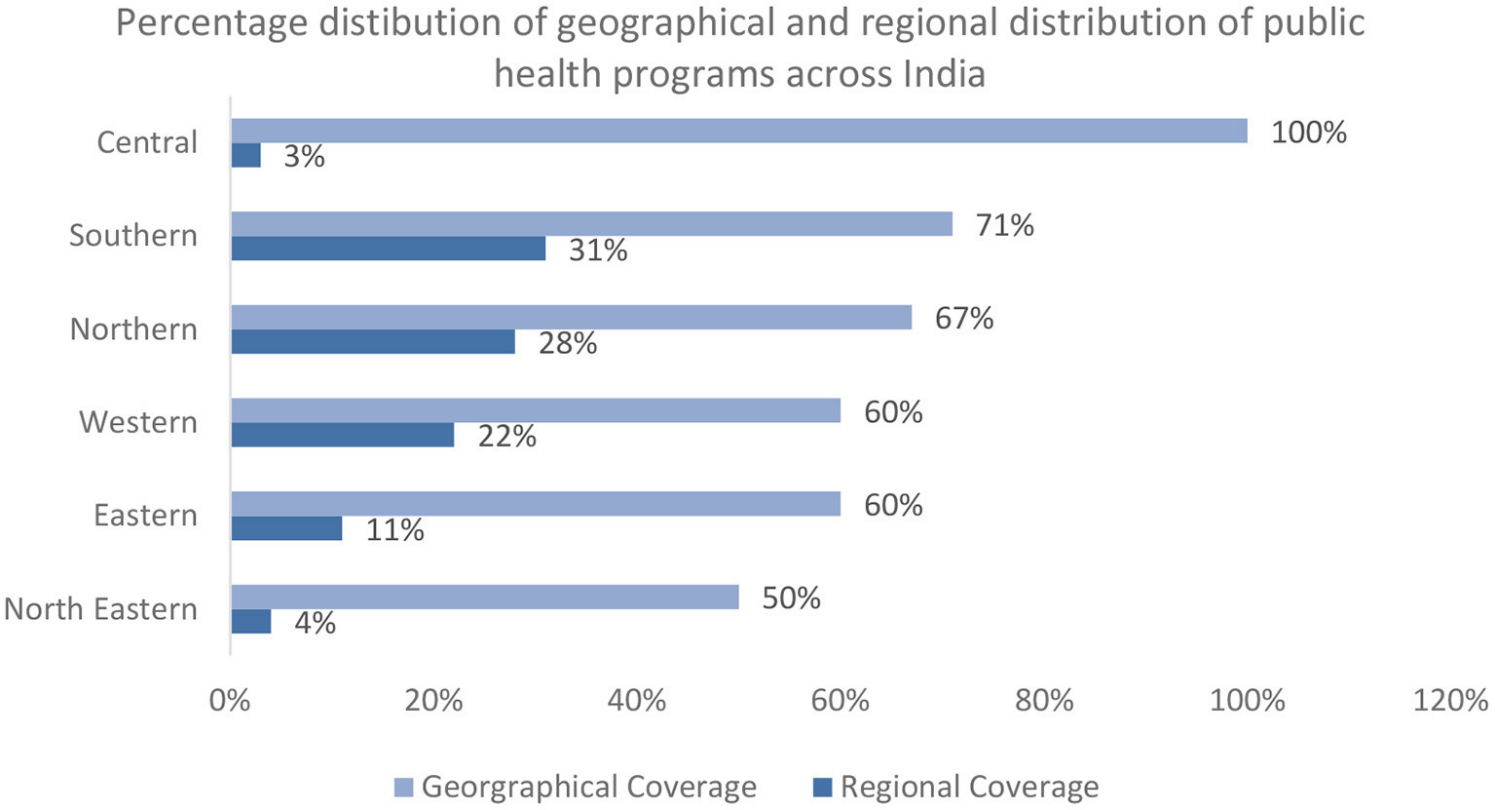
Percentage distribution of all public health programs across different geographical areas and regions of India.

All Indian states and UTs were categorized into six regions including North East, East, West, South, Central, and North Regional coverage was calculated by dividing the overall number of public health programme offerings in each region by the total number of public health programme offerings in India. Thirty-one percent (*n* = 36) of the public health programs are offered across the south, 28% (*n* = 32) across the north, and 22% (*n* = 26) across the west. The lowest number of public health program offerings were across the central and North East region of India (Figure 2). Geographical coverage was calculated by dividing the total number of states offering public health programs in each region by the total number of states in that region.

**Figure 3:**
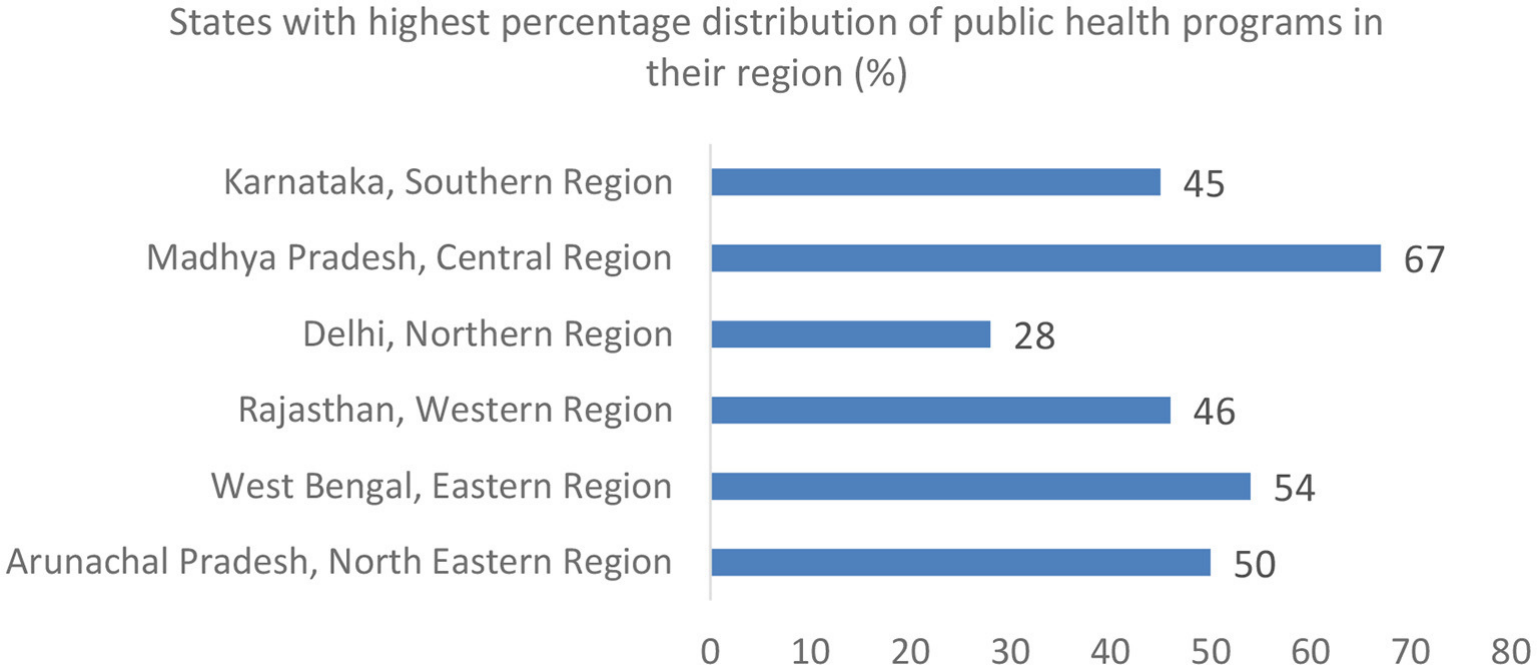
States offering most of the public health programs within their regions.

Madhya Pradesh in the Central, West Bengal in the East, Arunachal Pradesh in the North East, Karnataka in the South, Delhi in the North, and Rajasthan in the West were the states that offered most of the public health program offerings within their own regions (Figure 3).

#### Geographical coverage of program types across states and UTs of India

**Figure 4:**
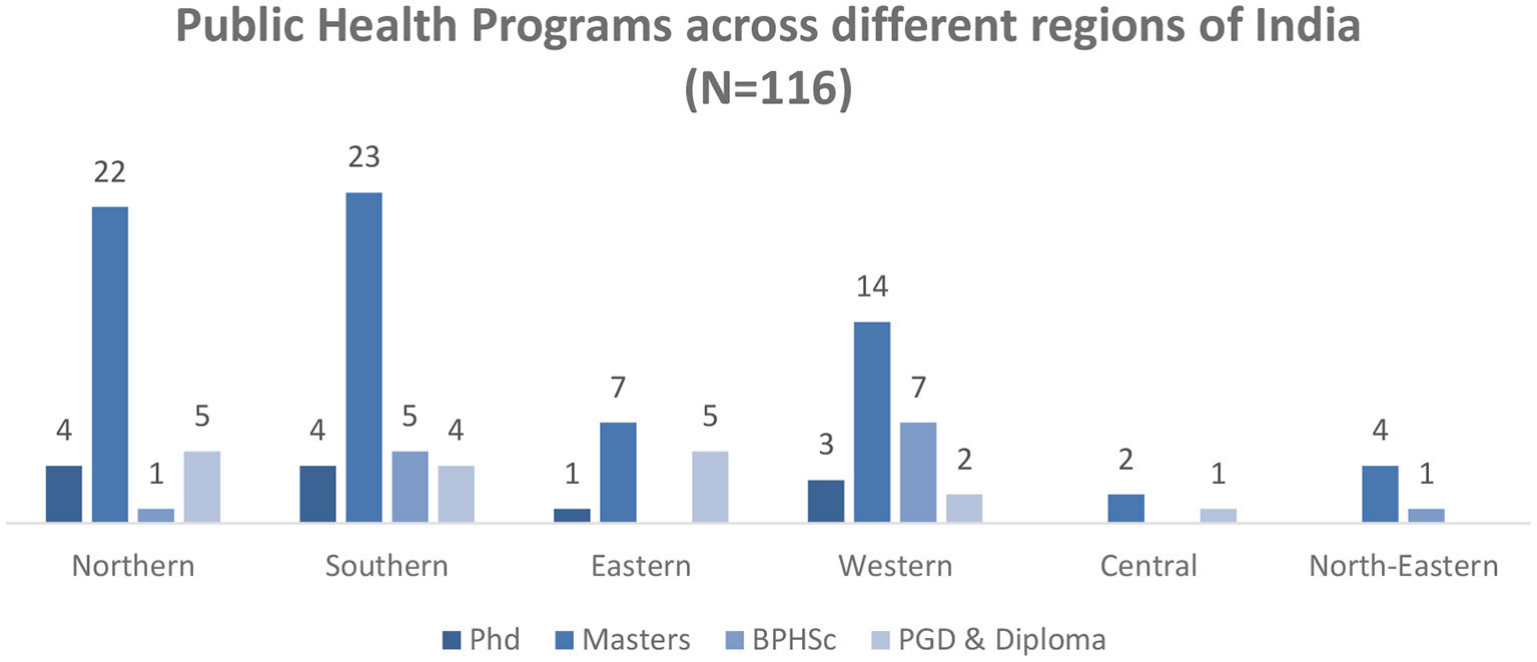
Distribution of public health programs across different regions in India.

**Figure 5:**
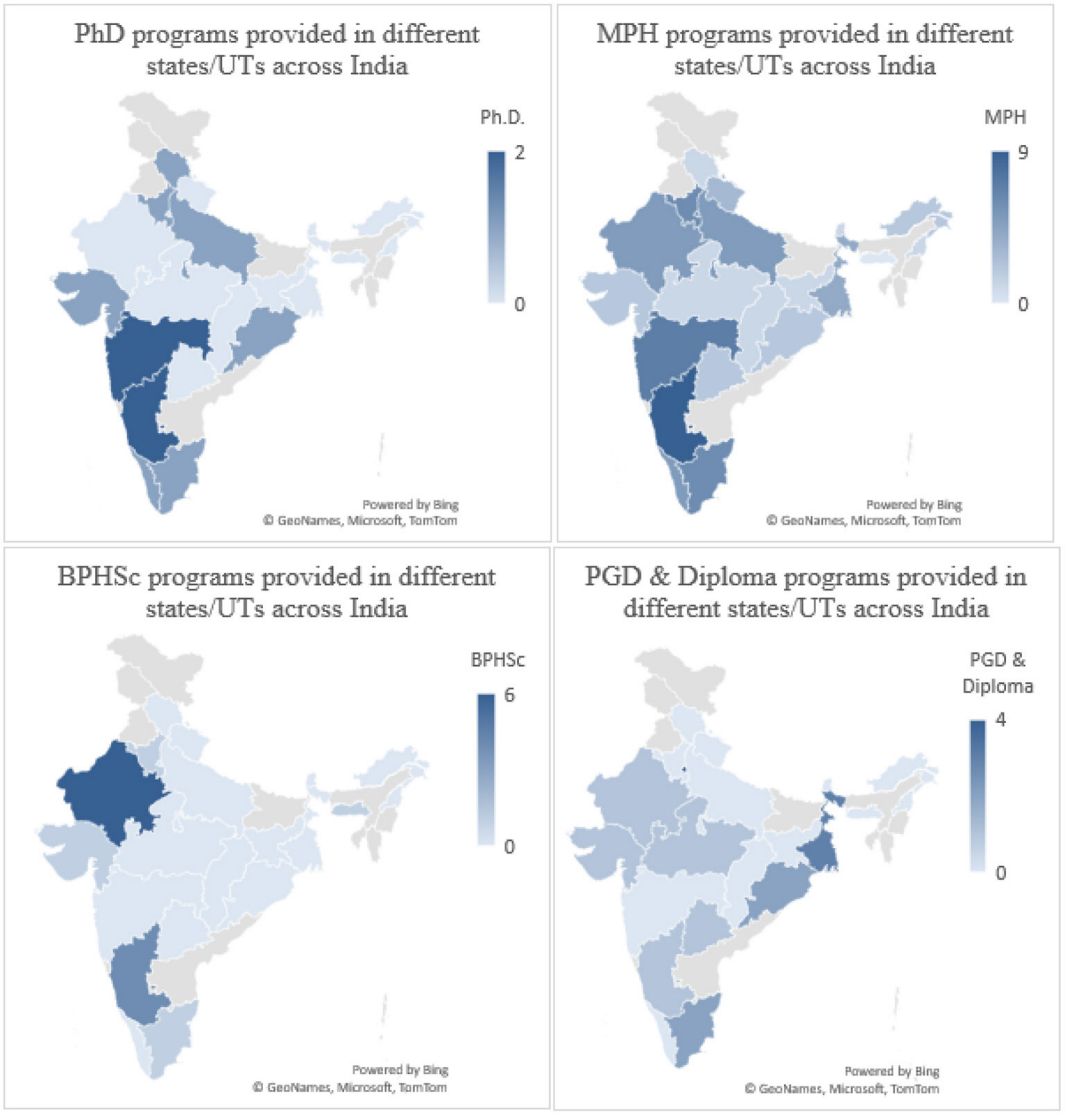
A comparison of all the different types of public health programs across different States/UTs in India.

Masters of Public Health programs were offered the most across all the regions of India (Figure 4). The states of Karnataka (South) (12%) Maharashtra (West) (10%), and Harayana (North) (8%) contributed the highest percentage toward the master of public health programs. Karnataka (17%) and Maharashtra (17%) also offered the highest percentage of doctoral programs in public health. Rajasthan (43%) and Karnataka (29%) offered the most bachelors in public health programs while the post-graduate diploma and diploma programs in public health were offered the highest across the states of Delhi (24%), Odisha (18%), West Bengal (12%) and Tamil Nadu (12%) (Figure 5).

### Analysis by programs

#### Doctorate in philosophy in public health (Ph.D.)

##### Regional distribution

Nearly twelve universities are providing Ph.D. programs in public health across India. The doctoral programs are offered mostly in the Southern (33%; *n* = 4) and Northern states (33%; *n* = 4) of India (Figure 5). Seventeen percent (*n* = 2) of the programs were been offered in the state of Maharashtra, Haryana, and Karnataka (Table 3).

**Table 3:** Ph.D. programs in public health in different states/UTs in India (*N* = 12).

**Parameter**	**Attributes**	***n*** **(%)**
Mode of delivery	Full-time and Part-time	5 (42)
	Full time	3 (25)
	Not mentioned	4 (33)
Type	Private	6 (50)
	Public	6 (50)
**States**	**Universities**	***n*** **(%)**
Odisha	AIPH University	1 (8)
Maharashtra	Savitribai Phule Pune University	1 (8)
	Tata Institute of Social Sciences	1 (8)
Karnataka	Institute of Public Health	1 (8)
	KLE Academy of Higher Education and Research	1 (8)
Uttar Pradesh	Amity University	1 (8)
Haryana	Amity University	1 (8)
	Post Graduate Institute of medical sciences	1 (8)
Tamil Nadu	SRM Institute of Science and Technology	1 (8)
Kerala	Sree Chitra Tirunal Institute for Medical Sciences and Technology, Trivandrum	1 (8)
Himachal Pradesh	Eternal University	1 (8)
Gujarat	Public health foundation of India	1 (8)

##### A.1 Total number of programs

Forty-two percent (*n* = 5) of the doctoral programs in public health are offered both part-time and full-time while 25% (*n* = 3) of them were offered only full-time. Thirty-three percent (*n* = 4) of the doctoral programs did not mention the modality of offering. Fifty percent (*n* = 6) of the doctoral programs in public health were offered by both public as well as private institutes.

##### A.2 Program fees

Of the 12 universities offering a doctoral program in public health, only half of them mentioned the fees details (50%; *n* = 6). Among the universities where the information was available, the average fee for each semester of the doctoral program is nearly INR 44,889 (Range: INR 14,833–INR 81,000), for the private universities the average fee is nearly INR 55,750 (Range: INR 42,000–INR 81,000) and whereas for the public universities the average fees are nearly INR 23,167 (Range: INR 14,833–INR 31,500).

##### A.3 Program format

Among the 12 universities, four are offering doctoral programs in public health in the fields of Biostatistics, Epidemiology, Health system research, Health promotion, health behavior, Global health, Migration, Urbanization, Public health, and health economics.

##### A.4 Program admission

Only two universities (*n* = 2, 17%) have mentioned the frequency of seat intake for doctoral programs in public health. Forty-two percent (*n* = 5) of the universities required for those applying from non-health backgrounds a minimum of 2–5 years of post-graduation work experience while those with a health background were required to have field experience, and prior experience in research, and teaching. Only two universities (*n* = 2, 17%) required publications as an essential criterion for admission to the doctoral program in public health. Fifty-eight percent of universities had an admission test (*n* = 7, 58%), 17% interview (*n* = 2, 17%), and 25% included both an admission test and an interview (*n* = 3, 25%) to be the criteria for admission. Eighty-three percent (*n* = 10) of the universities had a master's or equivalent degree awarded by an accredited university in India or abroad as the minimum academic qualification criteria for admission to the Ph.D. programs.

##### A.5 Additional details

None of the 12 universities presented any details on the website regarding course competencies the students will acquire. Four of the 12 universities offering doctoral programs in public health provided information related to the course curriculum indicating them to be designed as per the UGC choice-based credit system. However, only one out of these four universities shared a semester-wise Ph.D. program curriculum along with desired field/practical work. Only one university offers a fellowship for the Ph.D. program (Table 3).

#### Master's in public health (MPH)

##### Regional distribution

The majority of the master's in public health programs are offered in the southern region (*n* = 23, 32%) followed by nothern (*n* = 22, 30%), western (*n* = 14, 19%), eastern (*n* = 7, 10%), north-eastern (*n* = 4, 6%) and central region (*n* = 2, 3%). The geographical distribution of universities offering master's programs in public health highlights that in Karnataka (*n* = 9, 12%), Maharashtra (*n* = 7, 10%), and Tamil Nadu (*n* = 6, 8%) (Table 4).

**Table 4:** MPH programs delivered by the different states/UTs of India (*N* = 73).

**Parameters**	**Attributes**	***n*** **(%)**
Course duration (in years)	>2 years	2 (3)
	2 years	71 (97)
Type of universities	Public	19 (26)
	Private	54 (74)
States	Arunachal Pradesh	2 (3)
	Chhattisgarh	1 (1)
	Haryana	6 (8)
	Gujarat	2 (3)
	Uttar Pradesh	5 (7)
	Himachal Pradesh	1 (1)
	Jharkhand	1 (1)
	Karnataka	9 (12)
	Kerala	5 (7)
	Maharashtra	7 (10)
	Madhya Pradesh	1 (1)
	Nagaland	1 (1)
	Odisha	2 (3)
	Rajasthan	5 (7)
	Sikkim	1 (1)
	Tamil Nadu	6 (8)
	Telangana	2 (3)
	Uttarakhand	3 (4)
	West Bengal	4 (6)
Union territories	Universities	
	Chandigarh	2 (3)
	Delhi	5 (7)
	Pondicherry	1 (1)

##### B.1 Total number of programs

Of the 73 universities providing Master's programs in public health across India, 95% (*n* = 69, 95%) offered the program on campus. Nearly, Two-third (*n* = 54, 74%) of the public health programs were offered by private universities (Table 4).

##### B.2 Program fees

More than half of the programs (58%, *n* = 42) mentioned the program fee details on their website. Among the universities where the information was available, the average fee for the complete MPH program offered was INR 2,21,993 (Range: INR 5,865–INR 17,07,376). The average program fee for the private institute was INR 2,70,039 (Range: INR 34,150–INR17,07,376) and INR 1,08,233 (Range: INR 5,865–INR 2,50,000) for public universities.

##### B.3 Program format

More than half of the universities (*n* = 62, 85%) provided no information regarding specializations in public health. Only 15 percent of the programs listed (*n* = 11, 15%) mentioned their specializations ranging from the field of Epidemiology, Health Economics and Outcomes Research, Health Care Quality and Safety.

##### Admission requirements

Less than half of the institutes (38%, *n* = 28) mentioned seat intake for the masters in public health programs. Eighty-nine percent (*n* = 65) of the institutes offering Masters in public health programs required preferably a bachelor's degree with 50% marks in any discipline such as MBBS, BDS, AYUSH, B.Sc. Nursing, B. Pharma, BPT, BPH, B.Sc. Micro, B.Sc. Lab Tech (Med), B.Sc. For admission to the master's in public health degree, 15% of the universities (*n* = 11) required at least 1 year of work experience. Less than half of the institutions (42%, *n* = 31) required students to complete an internship as a course completion criterion. Twenty-five percent (*n* = 18) of colleges needed a written exam as part of the admissions process, 19% (*n* = 14) required interviews, and 14% (*n* = 10) required a combination of entrance test and interview.

##### Additional details

The course curriculum was presented on the institute website in 48% (*n* = 35) of the cases while <10% (*n* = 6) of universities provided details on the course competencies. The scholarship is offered by nine universities (*n* = 9, 12%) (Table 4).

#### Bachelors in public health sciences (BPHSc)

##### Regional distribution

Fourteen universities offering BPHSc programs, most of them are offered in the western region (*n* = 7, 50%) followed by the southern region (36% *n* = 5) (Table 5).

**Table 5:** Frequency of universities providing BPHSc in different states of India (*N* = 14).

**Parameters**	**Attributes**	***n*** **(%)**
Course duration (in years)	3 years	8 (57)
	Between 3 and 4 years	1 (7)
	4 years	4 (29)
	Not mentioned	1 (7)
Course category	BPH	10 (71)
	BSc	4 (29)
Type of universities	Public	3 (21)
	Private	11 (79)
**States**	**Universities**	***n*** **(%)**
Karnataka	Yenepoya University	1 (7)
	Rajiv Gandhi University of Health Sciences recognized by Govt. of Karnataka	2 (14)
	KLE Academy of Higher Education and Research	1 (7)
Tamil Nadu	SRM Institute of Science and Technology	1 (7)
Gujarat	Parul University	1 (7)
Rajasthan	Apex University	1 (7)
	Mahatma Jyoti Rao Phoole University	1 (7)
	Lachoo Memorial College of Science and Technology	1 (7)
	Career Point University	1 (7)
	Poornima University School of Public Health	1 (7)
	Singhania University	1 (7)
Meghalaya	Martin Luther Christian University	1 (7)
Haryana	Om Sterling Global University	1 (7)

##### C.1 Total number of programs

The majority of the bachelor's in public health programs were offered in Rajasthan (43%; *n* = 6) followed by Karnataka (29%, *n* = 4). More than half of these programs (79%, *n* = 11) were offered by private universities. The BPHSc course duration varied from 3 to 4 years. More than fifty percent of universities provided a complete course for 3 years (57%, *n* = 8) followed by 4 years (29%, *n* = 4) (Table 4). More than half of the programs are offered as Bachelor in Public Health (BPH) (71%, *n* = 10) while the remaining 29% (*n* = 4) of the programs are offered as BSc in Public Health.

##### C.2 Program fees

The average fee for a complete BPHSc program is around 1,56,000 (Range: INR 81,000–INR 2,40,000). The average fee of the BPHSc program for the public universities is INR 1,50,000 (Range: INR 0–INR 1,50,000) compared to private universities which is INR 1,57,000 (Range: INR 81,000–INR 2,40,000).

##### C.3 Program format

Almost all the BPHSc programs have been offered in a general format with no specializations and field of the study mentioned.

##### C.4 Program admission

Among all the 14 universities, 10 of them were admitted based on academic merit (*n* = 10, 71%). All the universities required educational qualification of higher secondary level or pre-university college in any stream, preferably science with physics, chemistry, and biology as main subjects as a minimum academic qualification for admission to the BPHSc programs. Fourteen percent of universities (*n* = 2) included interviews as one of the criterias for admission while 7% (*n* = 1) involved both an admission test and an interview for consideration of admission to the program.

##### C.5 Additional details

Fourteen percent (*n* = 2) of the universities provided details on course competencies while 35% (*n* = 5) of them provided information on course curriculum. Only one institute provided some form of scholarship (Table 5).

#### Postgraduate diploma in public health (PGDPh)

##### Regional distribution

The majority of the post-graduate diploma in public health programs are offered by universities in the western region (*n* = 2, 40%) followed by the southern region (*n* = 2, 29%).

##### D.1 Total number of programs

Of the 14 universities in different States/UTs of India, 29% (*n* = 4) of the universities in Delhi provide PGDPh programs followed by in eastern region Odisha and West Bengal (*n* = 2, 14%) each, and other states (Gujarat; Telangana; Madhya Pradesh; Chandigarh; Rajasthan, and Karnataka) offering one PGD program (*n* = 1, 7%) each. Among all universities offering PGD, (*n* = 2, 14%) were private and (*n* = 12, 86%) were public. Among the 12 public universities, (*n* = 7, 58%) provided courses as regular on-campus programs and (*n* = 4, 33%) in online mode whereas (*n* = 1, 8%) provided the course education in hybrid mode, that is, both online and on-campus. However, the two private universities run the course in off-campus (*n* = 1, 50%) and online (*n* = 1, 50%) mode, respectively (Table 6).

**Table 6:** Frequency of PGD programs provided by universities in the different states (*N* = 14).

**Parameters**	**Attributes**	***n*** **(%)**
Mode of delivery	On-campus	7 (50)
	Online	5 (36)
	Hybrid (On campus/Online/Distance)	1 (7)
	Off-campus	1 (7)
Type of universities	Public (On-campus/Online/Hybrid)	12 (86)
	Private	2 (14)
**States**	**Universities**	***n*** **(%)**
Odisha	Public health foundation of India	2 (14)
Gujarat	Public health foundation of India	1 (7)
Telangana	Public health foundation of India	1 (7)
West Bengal	West Bengal University of health sciences	1 (7)
	Government medical university	1 (7)
Madhya Pradesh	Indian Institute of skill development	1 (7)
Rajasthan	Singhania University	1 (7)
Karnataka	James Lind Institute	1 (7)
**Union territories**	**Universities**	
Delhi	Public health foundation of India	1 (7)
	Vocational Institution of Ministry of HRD, Government of India	1(7)
	Ministry of health and family welfare	1 (7)
	Indira Gandhi open university	1 (7)
Chandigarh	Post Graduate Institute of medical education and research	1 (7)

##### D.2 Program fees

The average fee for a complete PGDPh program offered by both private and public universities in different states is around one lakh (INR 1,02,240) (Range: INR 3,620–INR 2,75,000). For private universities, the average fee for a complete PGDPh program is around INR 32,750 (Range: INR 27,000–INR 38,500) and for public universities, the complete fee of the PGDPh program is nearly INR 1,07,551 (Range: INR 3,620–INR 2,75,000).

##### D.3 Program format

All of the PGD Ph programs are provided in a general format with no details on specializations and fields of study.

##### D.4 Program admission

Only 14% (*n* = 2) of the universities mentioned the frequency of admission intake. The minimum eligibility criteria required for admission to the PGDPh programs is to graduate in any discipline preferably MBBS, BDS, AYUSH, allied sciences, nursing, health science, natural science, or post-graduation in Social Sciences or an equivalent qualification. Additional qualifications include candidates who were working with the central, state, or local governments with at least 3 years of experience in the health sector, nursing staff, municipal corporation officers, and other professionals in health-related. More than half of the universities (86%; *n* = 12) offered admissions based on academic merit, entrance tests, interview performance, or both. Fifty-seven percent (*n* = 8) of the universities require at least 1 year of work experience.

##### D.5 Additional details

Of the 14 universities, 21% (*n* = 3) shared details on program competencies, while 36% (*n* = 5) mentioned the course curriculum (Table 6).

#### Diploma programs

##### Regional distribution

Two out of the three diploma programs are offered in the Southern part of India while one program is offered in the eastern region (Table 7).

**Table 7:** Frequency of diploma programs provided by universities in the different states (*N* = 3).

**Parameters**	**Attributes**	***n*** **(%)**
Type of universities	Public (On campus/Online/Hybrid)	1 (33)
	Private	2 (67)
**States**	**Universities**	***n*** **(%)**
Tamil Nadu	The Tamil Nadu Dr. M.G.R Medical University	1 (33)
Kerala	Sree Chitra Tirunal Institute of Medical Sciences and Technologies	1 (33)
West Bengal	West Bengal University of Health Sciences	1 (33)

Each diploma program is offered in the states of Tamil Nadu, Kerala, and West Bengal. Among all the three universities offering a Diploma in Public Health, two of the private universities are present in the state of Tamil Nadu and Kerala whereas the public university is in the state of West Bengal (Table 7).

##### E.2 Program fees

No fees were mentioned for any of the Diploma programs offered by either private or public universities.

##### E.3 Program format

Two out of the three programs listed on their website the duration of the diploma program in public health.

##### E.4 Program admission

None of the universities mentioned the frequency of seat intake in the diploma program. Different universities require different levels of prior work experience. e.g., the university in Kerala required at least 3 years of work experience for in-service candidates, and the university in West Bengal requires 1 year of continuous rotational internship training in any Medical College. Candidates' admission to one of the diploma programs offered by a University in West Bengal is based not only on academic merit but also on the entrance test conducted by the central or state government (if any) and the performance of the candidate in the interview.

##### E.5 Additional details

None of the Universities provided any information related to program competencies, course curriculum, or scholarships (Table 7).

Results of our analysis showed that the majority of Ph.D. programs in Public Health are offered in the states of Maharashtra, Karnataka, and Haryana while Masters in Public Health programs are offered highest in Karnataka, Bachelors in Public Health programs highest in Rajasthan, Post Graduate Diploma in Public Health program is highest in Delhi, and Diploma in Public Health program highest in the state of Tamil Nadu (Table 2).

## Discussion

The current study presents an overview of the various public health program offerings across various states and UTs of India. From the recent available literature, the unique institutes offering public health programs have increased from 59 institutes (8) to 84 unique institutes. Institutes offering Masters in Public Health have increased from 44 institutes in the years 2017 and 2018 (15) to 73 institutes according to our study in the year 2022.

Key findings of our study are: Of the 28 states and 8 UTs of India, 20 states and 3 UTs offered a public health-related program, the geographical concentration was highest in the southern states (*n* = 36, 31%) followed by the northern (*n* = 32, 28%) western (*n* = 26, 22%), eastern (*n* = 13, 11%), north-eastern (*n* = 5, 4%) and lastly, central regions of India (*n* = 3, 3%). Course competencies for Ph.D. and diploma programs are not mentioned by the universities, however, competencies were mentioned by one university offering a bachelor's program and 3 universities offering postgraduate programs. Of the programs studied, 35, 48, and 36% of institutes of doctoral, masters, and post-graduate programs respectively mentioned their course curriculum, other programs did not disclose their detailed curriculum. Few programs stated that their curriculum is as per the UGC choice-based credit system. Findings of our study show a lack of curriculum competencies as well as the mention of core concentration and cross-cutting fields on the website of the institutes (20, 21). A study by Miller et al.; reviewed the landscape of public health training and conducted a qualitative SWOT analysis among selected institutions. The study identified collaborations, lack of career pathways, and incentives as weaknesses and the strengths to be the tuition, innovation, and the available infrastructure for research (8). This finding correlates with our analyses, where program fees are kept affordable for quality education, this can be an advantage to increase student enrolment in public health programs. However, only 12% of MPH institutes and 7% of BPHSc institutes offered scholarships, this dearth of provision of scholarships and lack of public-private partnership is also reported among public health colleges across the globe as reviewed by Effa et al. (22) and Indian public health education by Scheiff et al. (23).

Findings of this review suggest (1) the need for additional public health incentive programs to be implemented to encourage public health workers to join local and state governments. (2) The requirement for well-trained public health workers should correspond to the new paradigm of place-led, which is at the heart of economic development strategy. (3) Aspirants in public health must be skilled in twentyfirst-century skills such as communication, informatics, leadership, policy, governance, and effective design and development of public health interventions. (4) To improve access to learning, public health initiatives should be equitably distributed throughout the country. The absence of educational facilities in certain regions can be attributed to per capita income. A good example from our analysis is the North Eastern, Eastern, and Central regions of India that have a low concentration of public health programs and notably nill doctorate programs; interestingly these states are reported to have the lowest per capita income and poor health indicators. There is a high disparity in health outcomes at regional and state levels. The backward states continue to have a high disease and mortality indices (24). (5) Aside from course-based curriculums, students should engage in experiential learning through internships, training, or research exposure to enhance their skills post their education.

The present review has the following limitations: Each program website was carefully searched for the enlisted variables. Owing to the differences in website content and lack of search engine optimization certain institute websites were not displayed in the results. The differences in content and format of the programs on respective websites resulted in some variables were not captured. The information gathered was unstructured on the official website pages, some information was found on the program page, some in a brochure, and some on the admissions page. On account of the dynamic nature of websites and updates in the content periodically there might be a difference in values extracted from August 2021 to November 2021 to date. The main drawback is the lack of a central counsel or regulating board for public health programs in India, there is no comprehensive data on all the institutes that offer public health courses in India.

The COVID-19 epidemic taught us the importance of an autonomous emergency system staffed by trained public health workers. While the past 2 years have profoundly raised public awareness of health, it has challenged the State, local and academic departments to substantially increase their capacity and contribute to the health workforce. To enable future preparedness, institutes across the country are to promptly work on capacity building for a future workforce that includes public health graduates (25).

The present review concurs with the view that public health education in India must now enhance health system performance by adapting core professional competencies to varied settings, which need to be revisited to deliver high-quality comprehensive public health services that are vital to improving population health equity (10). Our recommendations based on our observations are that the public health programs from universities and institutions can provide concise and clear information on student outcomes such as graduation rates, employment, career and placement opportunities, current alumni record, their roles, functions, and skills, and most importantly use of competencies gained in the field. With the increase in demand of job market coupled with the recent emphasis on public health cadre in states of India, career and placement services need to be a focus in public health program features. Additional information on the fee structure of different programs and on the salary structure of graduating students should be stated.

## Conclusion

The recent proposal by the government of India's Ministry of Health and Family Welfare (MoHFW) aimed to create a cadre across all states under the 2017 National Health Policy, this elucidates the urgent need to strengthen public health care delivery across India. To fulfill this, the workforce pool should be generated from institutes or universities having standardized programs with efficient curricula that equip them with necessary competencies. The public health programs across institutes have varied discrepancies in their deliverance and hence a synergistic effort across institutes can complement each other and equip the public health professional workforce in India.

## Author contributions

AJ and AG contributed to the conception and design of the study. MG collected and prepared the database. AJ, AB, and IVM performed the statistical analysis and interpretation. AJ, AB, and SSR prepared the draft of the manuscript. All authors were involved in manuscript revision and have approved the final version of the manuscript.

## Conflict of interest

The authors declare that the research was conducted in the absence of any commercial or financial relationships that could be construed as a potential conflict of interest.

## Publisher's note

All claims expressed in this article are solely those of the authors and do not necessarily represent those of their affiliated organizations, or those of the publisher, the editors and the reviewers. Any product that may be evaluated in this article, or claim that may be made by its manufacturer, is not guaranteed or endorsed by the publisher.
