# Supplementary material for: The current state of public health education in India: A scoping review
Source: Front Public Health. 2022 Nov 23;10:970617. doi: 10.3389/fpubh.2022.970617 (PMC9727389; doi:10.3389/fpubh.2022.970617)
Supplement: Supplementary file 1 [file Table_1.pdf]

Appendix-I

State	Institute/ University	Frequency
	**Doctorate Program**	
Gujarat	Public Health Foundation of India	1
Haryana	Amity University, Gurugram	1
Himachal Pradesh	Eternal University	1
Karnataka	1. Institute Of Public Health<br>2. KLE Academy of Higher Education and Research	2
Kerala	Sree Chitra Tirunal Institute for Medical Science and Technology	1
Maharashtra	1. Savitribai Phule Pune University<br>2. Tata Institute of Social Sciences	2
Odisha	AIPH University	1
Punjab	Post Graduate Institute of Public Health	1
Tamil Nadu	SRM Institute of Science and Technology	1
Uttar Pradesh	Amity University, Noida	1
	**Master's Program**	
Arunachal Pradesh	1. Arunodaya University<br>2. North East Frontier Technical University	2
Chandigarh	1. Panjab University<br>2. Chitkara University	2
Chattisgarh	AIIMS Raipur	1
New Delhi	1. Delhi Pharamaceuticals Sciences and Research University<br>2. Jawahar Lal Nehru University<br>3. Guru Gobind Singh Indraprastha University<br>4. Academy Of Scientific and Innovative Research<br>5. Jamia Hamdard University	5
Gujarat	1. Indian Institute of Public Health, Gandhinagar<br>2. Parul University	2
Haryana	1. Sushant University<br>2. G D Goenka University<br>3. SGT University	6

	4. OSGU (Om Sterling Global University)<br>5. Gurugram University<br>6. Amity University	
Himachal	Eternal University	1
Jharkhand	The Y.B.N University	1
Karnataka	1. JSS University<br>2. Manipal University<br>3. NIMHANS<br>4. NITTE Education Trust<br>5. Ramaiah University<br>6. Yenepoya University<br>7. Rajiv Gandhi University of Health Sciences<br>8. KLE University<br>9. Karnataka State Rural Development and Panchayat Raj University	9
Kerala	1. Shree Chitra Tirunal Institute for Medical Sciences and Technology<br>2. Amrita Vishwa Vidyapeetham<br>3. Kerala University of Health Sciences, Kottayam<br>4. Central University of Kerala<br>5. Kerala University of Health Sciences, Thiruvananthapuram	5
Madhya Pradesh	Krishna Institute of Medical Sciences	1
Maharashtra	1. Datta Meghe Institute of Medical Science<br>2. DY Patil University<br>3. MIT World Peace University<br>4. Pavara Institute of Medical Science<br>5. Savitribai Phule Pune University<br>6. Symbiosis University<br>7. Tata Institute of Social Science	7
Multiple Locations	Institute Of Clinical Research India	
Nagaland	The Global Open University	1
Odisha	1. AIPH University<br>2. Kalinga Institute of Industrial Technology	2
Pondicherry	Jawaharlal Institute of Postgraduate Medical Education and Research	1
Rajasthan	1. All India Institute of Medical Sciences<br>2. Career Point University<br>3. IIHMR: Johns Hopkins University	5

	4. Poornima University<br>5. University Of Technology	
Sikkim	SRM University	1
Tamil Nadu	1. SRM University<br>2. Indian Council of Medical Research and NIE<br>3. Christian Medical college (affiliated to Shree Chitra Tirunal Institute for Medical Sciences and Technology)<br>4. Sri Ramchandra Institute of Medical Sciences<br>5. Indian School of Business Management and Administration<br>6. Vinayaka Mission University	6
Telangana	1. Kaloji Narayana Rao University of Health Sciences, Warangal<br>2. University Of Hyderabad	2
Uttar Pradesh	1. Amity University, Noida<br>2. Sam Higginbottom University of Agriculture, Technology and Sciences<br>3. Noida International University<br>4. University Of Lucknow<br>5. Galgotias University	5
Uttarakhand	1. Sai Business and Media School, Sai Group of Institutions<br>2. All India Institute of Medical Sciences, (AIIMS)<br>3. Himalyan Garhwal University	3
West Bengal	1. Maulana Abul Kalam Azad University of Technology, Kolkata, West Bengal<br>2. Maulana Abul Kalam Azad University of Technology, Haldia, West Bengal<br>3. NSHM College Of Management and Technology, Kolkata<br>4. West Bengal University of Health Sciences	4
	**Bachelor's Program**	
Gujarat	Parul University	1
Haryana	Om Sterling Global University	1
Karnataka	1. Yenepoya University<br>2. Rajiv Gandhi University of Health Sciences: Padamshree Group of Institutions	4

	3. Rajiv Gandhi University of Health Sciences: Institute of Public Health and Centre for Disease Control<br>4. KLE Academy of Higher Education and Research	
Meghalaya	Martin Luther Christian University	1
Rajasthan	1. Apex University<br>2. Mahatma Jyoti Rao Phoole University<br>3. Lachoo Memorial College of Science and Technology<br>4. Career Point University<br>5. Poornima University School of Public Health<br>6. Singhania University	6
Tamil Nadu	SRM Institute of Science and Technology	1
	**Post Graduate Diploma Program**	
Chandigarh	PGIMER	1
New Delhi	1. Indian Institute of Public Health, PHFI<br>2. Indira Gandhi Open University<br>3. Vocational Institution of Ministry Of HRD,<br>4. Government Of India Ministry of Health and Family Welfare	4
Gujarat	Indian Institute of Public Health, PHFI	1
Karnataka	James Lind Institute	1
Madhya Pradesh	Indian Institute of Skill Development	1
Odisha	Indian Institute of Public Health, PHFI	1
Rajasthan	Singhania University	1
Telangana	Indian Institute of Public Health, PHFI	1
West Bengal	1. West Bengal University of Health Sciences<br>2. Government Medical University	2
	**Diploma Programs**	
West Bengal	West Bengal University of Health Sciences	1
Kerala	Shree Chitra Tirunal Institute of Medical Sciences and Technologies	1

Tamil Nadu	The Tamil Nadu Dr. M.G.R Medical University	1
